# Fruits and Vegetables Consumption and Associated Factors among In-School Adolescents in Five Southeast Asian Countries

**DOI:** 10.3390/ijerph9103575

**Published:** 2012-10-11

**Authors:** Karl Peltzer, Supa Pengpid

**Affiliations:** 1 HIV/STI and TB (HAST) Research Programme, Human Sciences Research Council, 134 Pretorius Street, Pretoria 0002, South Africa; 2 Department of Psychology, University of Limpopo, Turfloof Campus, Sovenga, Limpopo 0727, South Africa; 3 Department of Health System Management and Policy, University of Limpopo, Ga-Rankuwa Campus, Medunsa, Pretoria, 0204, South Africa; Email: supaprom@yahoo.com

**Keywords:** fruits, vegetables, adolescents, psychosocial correlates, health-compromising behaviours, Southeast Asian countries

## Abstract

The aim of the study was to assess the prevalence of fruits and vegetable consumption and associated factors among Southeast Asian in-school adolescents. Data were collected by self-report questionnaire from nationally representative samples (total 16,084) of school children aged 13 to 15 years in five Southeast Asian countries. Overall, 76.3% of the 13 to 15 year-olds had inadequate fruits and vegetables consumptions (less than five servings per day); 28% reported consuming fruits less than once per day and 13.8% indicated consuming vegetables less than once per day. In multivariable analysis, lack of protective factors and being physically inactive were associated with inadequate fruits and vegetable consumption, and sedentary behaviour and being overweight was protective of inadequate fruits and vegetable consumption. The results stress the need for intervention programmes aimed at increased consumption of fruits and vegetables, targeting proximal factors such as the family environment and distal factors by aiming at integrating other risk factors such as physical activity into health promotion among adolescents.

## 1. Introduction

High fruit and vegetable intake can promote health and prevent chronic diseases such as heart diseases and certain types of cancer [1,2]. The health benefits of fruits and vegetables seen in epidemiological studies are the main reasons for the recommended intake of at least 400 g of fruit and vegetables per day [3]. During adolescence several factors impact on chronic diseases: the development of risk factors, the tracking of risk factors throughout life, and the development of healthy or unhealthy habits that tend to stay throughout life [3]. Because of this, increasing fruit and vegetable consumption among children and adolescents is an important public health issue [4].

In spite of the importance of an adequate intake of fruits and vegetables during adolescence, large population groups, including children and adolescents in most Western countries [5,6], Asian countries [7,8,9,10,11], Costa Rica [12] and African countries [13] eat far less than the recommended amount of fruits and vegetables. The Health Behaviour in School-aged Children (HBSC) study 2001/2002 [5], which was conducted in 33 European and North American countries among 13 and 15-year-old students, showed that less than 50% of all young people reported eating vegetables and fruits on a daily basis. 

To develop effective interventions to increase fruit/vegetable intake, an understanding of etiological processes and the identification of potentially modifiable correlates is needed. Few studies have examined fruit/vegetable intake and its correlates among adolescents in Southeast Asian countries. Factors that have been found to be correlated with fruit and vegetable intake in previous studies of adolescents include age, gender, socioeconomic position, preferences, parental intake, parental modeling, family rules and parental encouragement and home availability/accessibility [4,14]. These correlates operate at a distal level (e.g., socioeconomic status), whereas others (e.g., parental practices, family rules, availability of fruits and vegetables) may operate at a more proximal level. Based on previous research it is hypothesized that distal level factors (e.g., socioeconomic status, culture or country) [4,15,16,17,18] and proximal level factors (family [4,14,19] and friends [20] related factors) will be correlated with fruits and vegetable consumption. This framework proposes that the more distal determinants of fruit and vegetable intake can be found in the cultural, physical and social environment and that they in turn influence the more proximal personal factors such as attitude, social influences and self-efficacy [21]. In addition, it is hypothesized that insufficient fruits and vegetable intake can be considered as a behaviour with underlying influences that are shared with other problem behaviours such as substance use [14], mental distress [19,22], and physical inactivity [4,23], which is consistent with the problem behaviour theory [24]. The aim of this study was to assess the prevalence of fruits and vegetable consumption and associated factors among 13 to 15 year-old in-school adolescents in five Southeast Asian countries.

## 2. Methodology

### 2.1. Description of Survey and Study Population

This study involved secondary analysis of existing data from the Global School-Based Health Survey (GSHS) from five Southeast Asian countries (India, Indonesia, Myanmar, Sri Lanka and Thailand). Details and data of the GSHS can be accessed at http://www.who.int/chp/gshs/methodology/en/index.html. For all countries national samples were included. The aim of the GSHS is to collect data primarily for students from age 13 to 15 years. A two-stage cluster sample design was used to collect data to represent all students in grades 6, 7, 8, 9, and 10 in the country. At the first stage of sampling, schools were selected with probability proportional to their reported enrollment size. In the second stage, classes in the selected schools were randomly selected and all students in selected classes were eligible to participate irrespective of their actual ages. School-aged youth completed a self-administered questionnaire within the selected school. Data collection was conducted by trained survey administrators during one regular class period. Student privacy was protected through anonymous and voluntary participation, and informed consent was obtained as appropriate from the students, parents and/or school officials.

### 2.2. Measures

The GSHS 10 core questionnaire modules address the leading causes of morbidity and mortality among children and adults worldwide: tobacco, alcohol and other drug use; dietary behaviors; hygiene; mental health; physical activity; sexual behaviors that contribute to HIV infection, other sexually-transmitted infections, and unintended pregnancy; unintentional injuries and violence; hygiene; protective factors and respondent demographics [25].

*Fruits and Vegetables Consumption and Hunger*: Fruits: “During the past 30 days, how many times per day did you usually eat fruit, such as ‘country specific examples’?”. Response options were 1 = I did not eat fruit during the past 30 days, 2 = less than one time per day, 3 = one time per day to 7 = five or more times per day. Vegetables: “During the past 30 days, how many times per day did you usually eat vegetables, such as ‘country specific examples’?”. Response options were 1 = I did not eat vegetables during the past 30 days, 2 = less than one time per day, 3 = one time per day to 7 = five or more times per day. The United States Department of Health and Human Services [15] recommends at least two daily servings of fruits and three daily servings of vegetables. Hunger: A measure of hunger was derived from a question reporting the frequency that a young person went hungry because there was not enough food at home in the past 30 days (response options were from 1 = never to 5 = always) (coded 1 = most of the time or always and 0 = never, rarely or sometimes). 

*Body Mass Index (BMI) Measurement and Overweight Classification*: Height and body weight were based on self-reports. BMI was calculated as weight/height^2^ (kg/m^2^). The international age-and gender-specific child BMI cut-points were used to define overweight and obesity [26]. These cut-points were derived from a large international sample using regression techniques by passing a line through the health-related adult cut-points at 18 years. Youth with BMI values corresponding to an adult BMI of ≥25.0 kg/m^2^ were classified as overweight. 

*Substance Use Variables*: Smoking cigarettes: During the past 30 days, on how many days did you smoke cigarettes? (response options were from 1 = 0 days to 7 = all 30 days) (coded 1 = 1 or 2 to all 30 days, and 0 = 0 days). Alcohol use: during the past 30 days, on how many days did you have at least one drink containing alcohol. Response options were from 1 = zero days to 7 = all 30 days; Coded 1 = 1 or 2 to all 30 days, and 0 = zero days. Drugs: During your life, how many times have you used drugs, such as glue, benzene, marijuana, cocaine, or mandrax? Response options were from 1 = zero times to 4 = ten or more times; Coded 1 = one or two to ten or more times, and 0 = zero times.

*Physical Activity*: Leisure time physical activity was assessed by asking participants: “Physical activity is any activity that increases your heart rate and makes you get out of breath some of the time. Physical activity can be done in sports, playing with friends, or walking to school. Some examples of physical activity are running, fast walking, biking, dancing, football. Do not include your physical education or gym class.” “During the past 7 days, on how many days were you physically active for a total of at least 60 minutes per day?” and “During a typical or usual week, on how many days are you physically active for a total of at least 60 minutes per day?” Leisure time sedentary behaviour was assessed by asking participants about the time they spend mostly sitting when not in school or doing homework: “How much time do you spend during a typical or usual day sitting and watching television, playing computer games, talking with friends, or doing other sitting activities.” Sedentary behaviour was defined as three or more hours sitting in a day.

*Psychological Distress*: Psychological distress was assessed with 5 items. Loneliness: “During the past 12 months, how often have you felt lonely?” (response options were from 1 = never to 5 = always) (Coded 1 = most of the time or always and 0 = never, rarely or sometimes). Suicide ideation: “During the past 12 months, did you ever seriously consider attempting suicide?” (response option was 1 = yes and 2 = no, coded 1 = 1, 2 = 0). No close friends:”How many close friends do you have?” (response options 1 = zero to 4 = three or more, coded 1 = one, 2–4 = zero.). Anxiety or worried: During the past 12 months, how often have you been so worried about something that you could not sleep at night? (response options were from 1 = never to 5 = always) (Coded 1 = most of the time or always and 0 = never, rarely or sometimes). Sadness: During the past 12 months, did you ever feel so sad or hopeless almost every day for two weeks or more in a row that you stopped doing your usual activities? (response option 1 = yes and 2 = no) (coded 1 = one, 2 = zero). A psychological index was created by adding up all five items, and recoding the sum into low = no psychological distress, medium = 1 item of psychological distress and high = 2 or more psychological distresses endorsed.

*Protective Factors*: Protective factors were assessed with five items on peer support at school, parental or guardian supervision, connectedness, and bonding. Peer support at school was assessed with the question “During the past 30 days, how often were most of the students in your school kind and helpful?” Parental or guardian supervision “During the past 30 days, how often did your parents or guardians check to see if your homework was done”? Parental or guardian connectedness “During the past 30 days, how often did your parents or guardians understand your problems or worries?” and Parental or guardian bonding “During the past 30 days, how often did your parents or guardians really know what you were doing with your free time?” Response options to these questions were from 1 = never to 5 = always, coded 1 = never or rarely and 0 = sometimes to always. All four protective factor items were added up, and recoded into low, medium and high lack of protective factors.

### 2.3. Data Analysis

In order to compare study samples across countries each country sample was restricted to the age group 13 to 15 years, younger and older participants were excluded from the analyses. For each country, the overall response rates ranged from 81 to 95% (see Table 1).

**Table 1:** Sample response rate and age distribution of students surveyed; GSHS 2006–2008.

Country	Survey year	Overall response rate ^1^	Age groups in years (%)			Boys in final sample	Mean age of final sample
		%	13 years	14 years	15 years	%	Mean
1. India	2007	84	2,017 (29.9)	2,654 (38.4)	2,080 (31.7)	57.9	14.0
2. Indonesia	2008	93	1,072 (33.2)	1,253 (45.2)	542 (21.6)	49.5	13.9
3. Myanmar	2007	95	585 (37.1)	628 (34.3)	770 (28.6)	50.0	13.9
4. Sri Lanka	2007	89	894 (38.9)	844 (37.3)	522 (23.8)	50.4	13.8
5. Thailand	2008	93	841 (37.1)	871 (36.2)	511 (26.7)	49.2	13.9

^1^ Overall response rate, the product of school and the student response rate, refers to the entire sample including those students outside the targeted age range of 13 to 15 years.

Data analysis was performed using STATA software version 10.0 (Stata Corporation, College Station, TX, USA). This software has the advantage of directly including robust standard errors that account for the sampling design, *i.e.*, cluster sampling owing to the sampling of school classes. Associations between distal factors (sociodemographic variables, hunger), proximal factors (protective factors) and other risk behaviour (mental distress, physical inactivity, substance use and overweight) among school children were evaluated calculating odds ratios (OR). Logistic regression was used for evaluation of the impact of explanatory variable for inadequate fruits and vegetables (<5 servings per day) (binary dependent variable). In the analysis, weighted percentages are reported. The reported sample size refers to the sample that was asked the target question. The two-sided 95% confidence intervals are reported. The *p*-value less or equal to 5% is used to indicate statistical significance. Both the reported 95% confidence intervals and the *p*-value are adjusted for the multistage stratified cluster sample design of the study.

## 3. Results and Discussion

### 3.1. Sample Characteristics and Fruits and Vegetable Consumption

The total sample included 16,084 school children aged 13 to 15 years from five Southeast Asian countries. There were slightly more male (51.2%) than female (48.8%) school children. In all most children 76.3% consumed less than the recommended five servings of fruits and/or vegetable, this was the same among boys and in girls. Twenty-eight percent of the participants reported consuming less than once fruits per day and 13.8% indicated consuming less than once vegetables per day. The mean number of fruits consumed per day was 1.3, far below the recommended two fruits a day target. The mean number of vegetable consumption was 1.9, also far below the recommended three vegetables a day target. The mean daily servings of fruits and/or vegetables per day was 3.2, far below the recommended five servings a day target. Indian and Myanmar school children had the most insufficient. In terms of mean daily servings of fruits and/or vegetables, Thai school children had the highest consumption (3.7), followed by Indonesian (3.2) and Sri Lankan (3.1) school children, and the lowest consumption was among Myanmar (2.9) and Indian (3.0) school children (see Table 2).

**Table 2:** Details of participating country samples included in the analyses (age 13–15 years only) and fruits and vegetable consumption; GSHS 2006–2008.

Country	Total	Mean daily servings of			Fruits <1 or more	Vegs <1 or more	Fruits & Vegs <5
		fruits	vegetables	fruits + vegetables			
	N	M (SD)	M (SD)	M (SD)	%	%	%
1. India	6,751	1.1 (1.0)	1.9 (1.1)	3.0 (1.6)	28.4	10.2	85.1
2. Indonesia	2,867	1.4 (1.3)	1.8 (1.3)	3.2 (2.1)	30.3	16.7	75.2
3. Myanmar	1,983	1.2 (1.1)	1.7 (1.2)	2.9 (1.9)	23.7	10.8	83.3
4. Sri Lanka	2,260	1.2 (1.3)	1.9 (1.3)	3.1 (2.1)	34.5	15.3	77.1
5. Thailand	2,223	1.6 (1.4)	2.1 (1.4)	3.7 (2.3)	24.1	13.0	67.1
Total	16,084	1.3 (1.4)	1.9 (1.3)	3.2 (2.1)	28.0	13.7	76.3

### 3.2. Inadequate Fruits and Vegetable Consumption and Its Relationship with Distal and Proximal and Other Risk Factors

In univariate analysis lack of protective factors and being physically inactive were associated with inadequate fruits and vegetable consumption and substance use (alcohol use and smoking), sedentary behaviour and being overweight or obese were protective of inadequate fruits and vegetable consumption. In multivariable analysis lack of protective factors (OR = 1.45, 95% CI = 1.16–1.81) and being physically inactive (OR = 1.34, 95% CI = 1.10–1.63) were associated with inadequate fruits and vegetable consumption and sedentary behaviour (OR = 0.77, 95% CI = 0.65-0.92) and being overweight (OR = 0.76, 95% CI = 0.61–0.96) were protective of inadequate fruits and vegetable consumption (see Table 3).

**Table 3:** Univariate and multivariate logistic regression of inadequate fruits and vegetable consumption, GSHS 2007–2008.

	N (%)	Crude OR (CI 95%)	Adjusted OR (CI 95%)
**Sociodemographic variables**			
*Gender*			
Female	6,159 (76.3)	Ref	Ref
Male	6,468 (76.3)	1.00 (0.90–1.12)	0.94 (0.82–1.09)
*Age*			
13 years	4,188 (75.7)	Ref	Ref
14	4,932 (76.6)	1.05 (0.94–1.17)	1.01 (0.87–1.18)
15 years	3,546 (76.5)	1.04 (0.91–1.20)	0.95 (0.81–1.13)
Went hungry	519 (72.6)	0.82 (0.62–1.07)	---
**Protective factors**			
*Lack of protective factors*			
Low	4,714 (74.1)	Ref	Ref
Medium	3,050 (76.2)	1.14 (1.02–1.28) *	1.20 (1.04–1.38) *
High	2,060 (80.9)	1.48 (1.23–1.75) ***	1.45 (1.16–1.81) **
**Other risk factors**			
*Mental distress*			
Zero	4,771 (74.8)	Ref	---
One	1,437 (73.0)	0.91 (0.78–1.06)	
Two or more	592 (72.4)	0.88 (0.70–1.11)	
*Substance use*			
Current alcohol use	281 (68.1)	0.73 (0.56–0.94) *	0.81 (0.57–1.75)
Current smoking	414 (69.3)	0.68 (0.54–0.86) **	0.80 (0.56-1.15)
Ever drugs	116 (72.2)	0.92 (0.56-1.50)	---
*Physical activity*			
Less than 60 minutes daily	9,925 (76.9)	1.24 (1.08–1.42) **	1.34 (1.10–1.63) **
Sedentary behaviour	3,184 (72.5)	0.75 (0.67–0.85) ***	0.77 (0.65–0.92) **
*Overweight *	717 (69.7)	0.69 (0.57–0.83) ***	0.76 (0.61–0.96) *

*** *P* < 0.001; ** *P* < 0.01; * *P* < 0.05.

## 4. Discussion

The current study explores fruits and vegetable consumption and its correlates among in-school adolescents from nationally representative samples in five Southeast Asian countries. Overall, low fruits and vegetable consumption were reported, as 76.3% of the sampled population consumed less than the recommended five servings of fruits and/or vegetable, 28% reported consuming less than once fruits per day and 13.8% indicated consuming less than once vegetables per day. Studies from other low and middle income countries and high income countries seem to confirm low fruits and vegetable consumption levels among adolescents, e.g., in seven African countries 77.5% of adolescent learners [13] and 78.6% of American high school students [27] had eaten less than five servings of fruits and vegetables per day. It is possible that in the study countries undergoing health transition in such a way that staples and side dishes are being replaced by diets containing higher proportions of fats and animal meat, and less vegetables and fruits [28,29,30,31]. On the whole Asia falls with 183.4 kg far above the 146 kg fruits and vegetable availability per person per year recommended by WHO/FAO [32]. 

This study found cross-national variations in the prevalence of fruits and vegetable consumption. In terms of mean daily servings of fruits and/or vegetables Thai school children had the highest consumption (3.7), followed by Indonesian (3.2), Sri Lankan (3.1), Indian (3.0) and Myanmarian (2.9) school children. Possible reasons for such country differences could be fruits and vegetable availability, income and urbanization rate. Availability data on fruits and vegetables in the study countries seem to confirm a relationship between consumption and availability for Thailand and Sri Lanka but not for India, Indonesia and Myanmar (see Table 4). The WHO [32] notes that fruits and vegetable consumption increase with income and with urbanization. Yet, data from the World Health Survey (WHS) show that for men and women combined (18–99 years), the poorest quintile had the highest prevalence of low fruit and vegetable consumption and although urbanicity was not associated overall with low fruit and vegetable consumption, urban and rural differences were significant for eleven countries; urban residents had a higher risk of low fruit and vegetable consumption in Bangladesh and Philippines [33]. 

In our study Thai school children had the highest consumption of fruits and vegetables, which may be due to larger availability of fruits and vegetables and greater income (also found by Satheannoppakao *et al.* [34] in Thailand) than other study countries. In addition, among the Asian study countries, only in Thailand the government has started a number of school initiatives related to increased promotion of fruits and vegetables [35]. Kosulwat [28] found that one of the positive behavioural changes in Thailand was the increased intake of vegetables and fruits since 1986. On the other hand, low consumption of fruits and vegetables in India [36], e.g., only 39.4% of children were having fruits daily [37] and Indonesia, with 94% of the people above the age of 10 years eating vegetables and/or fruits less than the minimum 5 portion per day in a 7-day period has been found in other studies [38]. Moreover, different social, cultural and livelihood factors, such as food culture, nutrition knowledge, dietary attitudes and habits, level of farm activities, and marketing strategies in the study countries can also influence the levels of fruit and vegetable consumption [32,34,39,40]. 

**Table 4:** Country comparison of fruits and vegetable consumption and associated indicators, GSHS 2007–2008.

	Country				
	India	Indonesia	Myanmar	Sri Lanka	Thailand
Mean daily servings of fruits and/or vegetables (13–15 years) (2007–2008) ^1^	3.0	3.2	2.9	3.1	3.7
Prevalence of low fruit and vegetable consumption (13–15 years) (2007–2008) ^1^	85.1	75.2	83.3	77.1	67.1
Prevalence of low fruit and vegetable consumption (18–99 years) [33] (2002–2003) ^1^	74.2	58.6 [41]	83.7	67.6	73.4 [34]
Availability of fruits *per capita* per day in grams (2002) [41]	103.3	98.6	82.2	114.5	240.5
Availability of vegetables *per capita* per day in grams (2002) [41]	190.4	76.4	183.0	91.8	115.3
Mostly or always went hungry (13–15 years) (2007–2008) ^1^	3.5	5.8	2.6	6.5	3.2
% of under-fives underweight [43] (2000–2009) ^1^	43.5	19.6	29.6	21.6	7.0
Gross National Income *per capita* (US$) [42] (2009) ^1^	3,250	3,720	…	4,720	7,640
Urbanization (%) [42] (2009) ^1^	30	53	33	15	34

^1 ^Year of data collection.

This study found in concurrence with other studies that lack of protective factors such as family-related factors (parental style; lack of caregiver bonding, connectedness and supervision) [4,14,19], and friends-related factors (lack of peer support at school) [20] were associated with inadequate fruits and/or vegetable intake. Further, consistent with problem behaviour theory the study found that physical inactivity [23] was associated with inadequate fruits and/or vegetable consumption. Sedentary leisure time behaviour (fewer hours sitting in a day) was in this study associated with inadequate fruits and/or vegetable consumption, while other studies found a positive association between hours watching TV and inadequate fruits and/or vegetable consumption [4]. Finally, being overweight or obese was in this study found to be protective of inadequate fruits and vegetable consumption. Alinia *et al.* [43] reviewed that the majority of the evidence points towards a possible inverse association between fruit intake and overweight, and Ledoux *et al.* [44] found that an inverse relationship between fruit and vegetable intake and adiposity among overweight adults appears weak and that this relationship among children is unclear. They suggest further research to clarify the effects of fruits and vegetable consumption on adiposity. Unlike other studies [4,5,15,16,17,18,19,22], this study did not find any association between distal factors such as gender (boys), age (older age), socioeconomic position (went without food), substance use (smoking, alcohol use), mental distress and inadequate fruits and/or vegetable consumption. 

## 5. Conclusions

The cross-national data found inadequate fruits and vegetable consumption in five Southeast Asian countries, pointing to the conclusion that programmes are needed to improve fruit and vegetable consumption of the adolescent population. Interventions to improve health-related behaviours should be tailored to the most important determinants or mediators of these behaviours including targeting proximal factors such as the family environment for the promotion of healthy eating behaviours among adolescents. The risk factors identified were consistent with the problem behaviour theory in which inadequate fruits and/or vegetable consumption are shared with other problem behaviours such as physical inactivity, which indicates that health promotion programmes should be broadened to include these factors collectively in health interventions for adolescents.

### 5.1. Limitations of the Study

This study had several limitations. Firstly, the GSHS only enrolls adolescents who are in school. School-going adolescents may not be representative of all adolescents in a country as the occurrence of fruits and vegetable consumption may differ between the two groups. As the questionnaire was self-completed, it is possible that some study participants may have miss reported either intentionally or inadvertently on any of the questions asked. Intentionally miss reporting was probably minimized by the fact that study participants completed the questionnaires anonymously. Further, the assessment of correlates of fruits and vegetable consumption was limited and other factors such as preferences, parental intake, parental modeling, family rules and parental encouragement, home availability/ accessibility, school level factors such as nutrition education, availability and policy of healthy and unhealthy foods, community and or neighbourhood level factors, and national level factors such as price levels, policy, guidelines, supply, and exposure to mass media and commercials should be included in other studies among adolescents [4,14,19]. Furthermore, this study was based on data collected in a cross-sectional survey. We cannot, therefore, ascribe causality to any of the associated factors in the study. Prospective studies are required to follow up fruits and vegetable consumption and a more comprehensive list of influencing factors.
